# Photo-Scanning Capacitance Microscopy and Spectroscopy Study of Epitaxial GaAsN Layers and GaAsN P-I-N Solar Cell Structures

**DOI:** 10.3390/nano15141066

**Published:** 2025-07-09

**Authors:** Adam Szyszka, Wojciech Dawidowski, Damian Radziewicz, Beata Ściana

**Affiliations:** Faculty of Electronics, Photonics and Microsystems, Wrocław University of Science and Technology, Janiszewskiego 11/17, 50-327 Wrocław, Poland; wojciech.dawidowski@pwr.edu.pl (W.D.); damian.radziewicz@pwr.edu.pl (D.R.); beata.sciana@pwr.edu.pl (B.Ś.)

**Keywords:** scanning capacitance microscopy, SCM, EC-V, GaAsN, dilute nitrides, light spectroscopy, solar cell

## Abstract

This work presents a novel approach to investigating epitaxial GaAsN layers and GaAsN-based p-i-n solar cell structures using light-assisted scanning capacitance microscopy (SCM) and spectroscopy. Due to the technological challenges in growing high-quality GaAsN with controlled nitrogen incorporation, the epitaxial layers often exhibit inhomogeneity in their opto-electrical properties. By combining localized cross-section SCM measurements with wavelength-tunable optical excitation (800–1600 nm), we resolved carrier concentration profiles, internal electric fields, and deep-level transitions across the device structure at a nanoscale resolution. A comparative analysis between electrochemical capacitance–voltage (EC-V) profiling and photoluminescence spectroscopy confirmed multiple localized transitions, attributed to compositional fluctuations and nitrogen-induced defects within GaAsN. The SCM method revealed spatial variations in energy states, including discrete nitrogen-rich regions and gradual variations in the nitrogen content throughout the layer depth, which are not recognizable using standard characterization methods. Our results demonstrate the unique capability of the photo-scanning capacitance microscopy and spectroscopy technique to provide spatially resolved insights into complex dilute nitride structures, offering a universal and accessible tool for semiconductor structures and optoelectronic devices evaluation.

## 1. Introduction

Dilute nitrides, such as GaAsN, GaSbN, GaInNAs, and GaInNAsSb are examples of materials with desirable physical properties for the application of optoelectronic devices, but their exploration has been limited by technological difficulties in the growth of high-quality structures. The specific feature of this semiconductor compound is that nitrogen’s high electronegativity (3.04 in Pauling scale) and small atomic radius of 65 pm compared to that of As (2.18; 115 pm) significantly reduces its bandgap (150 to 200 meV) when a small amount of nitrogen (e.g., 1%) is incorporated into GaAs at the arsenic site [1,2]. This bandgap reduction is advantageous for high-performance optoelectronic devices operating in the near-infrared region, such as infrared-extended GaAs-based solar cells, photodetectors, laser diodes, and modulators for fiber-optic communication systems [3].

The ability to tune the bandgap of GaAsN to emit and detect in the wavelength of the second and third optical windows is of great importance for fiber telecommunication systems. Traditionally, devices operating at these wavelengths require expensive and complex indium phosphide substrates. GaAsN allows for fabrication directly on mature, cost-effective GaAs substrates, leading to reduced manufacturing costs, improved integration, and enhanced scalability. In photovoltaics, diluted nitrides play a critical role in multi-junction solar cells by enabling the fabrication of sub-cells with tailored bandgaps around 1.0–1.2 eV, necessary for optimal sun spectrum utilization. Employing GaInNAsSb bottom junctions in triple-junction cells has led to efficiencies of 43.5% at 25 °C [4]. Development in the field of GaAsN-based devices is carried out not only by optimizing the design and technology of planar devices, but also by developing new, unconventional systems, such as GaAsN/GaAsN:H quantum dot single-photon emitters [3], nanowire lasers based on a GaAs/GaNAs core/shell structure [5], and III-N-V core-multi-shell nanowire high-efficiency intermediate-band photovoltaic cells [6].

However, nitrogen incorporation in GaAs is challenging, as it leads to deterioration in the crystal (lattice parameter reduction, biaxial tensile stress induced by nitrogen atoms presence), optical (absorption redshift and decrease in photoluminescence signal) [7], and electrical (splitting of conduction band edge into two subbands) [8] properties due to nitrogen-related point defects, split configurations [9], non-uniform N atom distribution [10], and compositional fluctuations [11]. Therefore, controlling the nitrogen content without phase separation remains a technological challenge due to its low incorporation probability and sensitivity to growth conditions.

In this work, our innovative photo-SCM microscopy and spectroscopy method is used for the first time to measure cross-sections of multilayer semiconductor structures. So far, we have used this methodology to examine the influence of different types of surface defects on local electrical property variations in AlGaN/GaN/Si heterostructures [12]. However, these investigations were carried out in a typical AFM measurement configuration, where the upper surface of the samples was scanned and only near-surface changes in material properties could be determined. Here, the measurement results of sample cross-sections will be presented, allowing for precise in-depth analysis of the properties of p-i-n junction cell structures with GaAaN base layers and providing detailed insight into the internal properties of the diluted nitride semiconductor layers themselves. Understanding the basic electrical properties of the epitaxial structure is crucial for the fabrication of optoelectronic devices.

This manuscript can be summarized as follows. The introduction presents the technology and construction of the two GaAsN-based samples used in the research, including a p-i-n solar cell structure and a reference GaAsN epilayer. Then, the applied measurement methods are described, with special emphasis on the developed photo-SCM technique. The photoluminescence method was used to determine the optical properties, while the electrochemical capacitance–voltage method was used to determine the electrical properties of the structures. Photo-SCM measurements of the cross-sections of the GaAsN-based structures were performed in two modes: (1) obtaining SCM profile lines of the samples under excitation with defined photon energy and (2) measuring the SCM signal vs. photon energy spectra at specific locations on the sample. The existence of many energy transitions associated with defects and the structural inhomogeneity of the GaAsN layers was demonstrated. Based on the measurements using the photo-SCM, photoluminescence, and capacitance-voltage methods, as well as previous X-ray diffraction measurements, partial identification and analysis of these transitions was performed.

## 2. Materials and Methods

Two samples were investigated and grown using the atmospheric pressure metal–organic vapor phase epitaxy (AP-MOVPE) technique on (100) GaAs substrates (n-type for the solar cell structure and p-type for the GaAsN test epilayer), using a horizontal AIX 200 R&D reactor and high-purity hydrogen as the carrier gas. Arsine (10% mixture in H_2_), trimethylgallium (TMGa), trimethylaluminium (TMAl), and an organic source of nitrogen tertiarybutylhydrazine (TBHy) were used as growth precursors, while diethylzinc (DEZn) and silane SiH_4_ (20 ppm in H_2_) served as dopant sources. Details about the growth conditions are presented in Table 1.

**Figure 1:**
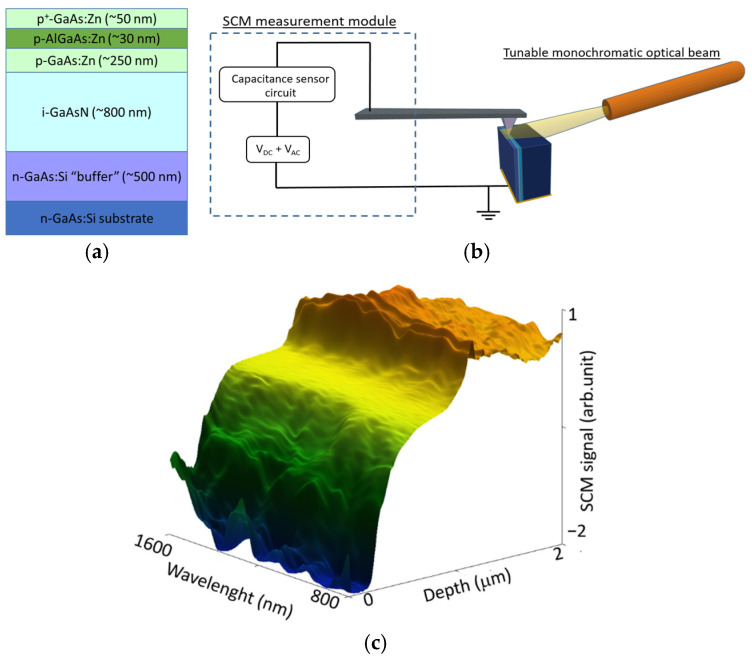
Schematic cross-section of GaAsN p-i-n solar cell structure (**a**); SCM measurement scheme with simultaneous sample illumination (**b**); an example of the resulting measurement graph, showing the SCM signal profiles across the sample for the following wavelength changes (**c**).

The schematic cross-section of the examined GaAsN-based solar cell with a p-i-n junction is shown in Figure 1. The presented epitaxial structure consists of an 800 nm undoped GaAsN base layer deposited on 500 nm silicon-doped GaAs. The base is followed by a 250 nm zinc-doped GaAs emitter, a thin zinc-doped AlGaAs optical window, and a highly zinc-doped GaAs contact layer. The second investigated sample with a test GaAsN epilayer of about 820 nm was deposited on a p-type GaAs buffer with a thickness of 500 nm. The growth temperature was changed from 575 °C for GaAsN to 700 °C for GaAs and AlGaAs [13,14]. The thickness and composition of the AlGaAs were determined to be about 30 nm and 50% of Al, respectively.

Optical characterization was performed with photoluminescence (PL) measurements at 90 K and room temperature using the Oxford Instruments OptistatDN-V cryostat, the InGaAs Hamamatsu C9913GC, StellarNet Blue-Wave UVIS single-channel detectors, and the 405 nm continuous wave (CW) laser RGB Lasersystem with maximal optical power of 100 mW.

Electrically active carrier concentration profiles were measured with the electrochemical capacitance–voltage method (E-CV) using the PN 4300 Biorad system with Tiron electrolyte. Electrochemical capacitance–voltage profiling is an extension of the classical C-V method, where the carrier type and its concentration are measured using the properties of the Schottky-like semiconductor–electrolyte junction. The junction area was precisely determined using the sealing ring. The junction capacitance was measured at a constant DC bias, with decreases in capacity C indicating n-type conductivity and decreases indicating p-type conductivity with increasing bias voltage V. In classical C-V measurements, analysis is performed at the end of the depletion zone; therefore, the measurement depth is limited by the depletion width. After each electrochemical C-V measurement, a selected depth of the semiconductor material was etched using electrochemical dissolution in the electrolyte, and the capacitance measurements were repeated. This omitted the depth limitation of the C-V approach. However, the etching process was destructive to the samples and produced small holes. Despite this, the nanometer scale-controlled etching of the material and subsequent C-V measurements enabled the construction of a free carrier distribution profile with regard to depth.

The scanning probe microscopy system used in this work is based on a commercial Bruker Multimode Nanoscope V Atomic Force Microscope (AFM) equipped with a scanning capacitance module, and expanded using an external, custom-made sample illumination setup.

In the scanning capacitance microscopy (SCM) technique, an electrically conductive AFM probe scans the sample surface in constant contact mode. When a thin isolating layer exists on the semiconductor surface (such as a native oxide, as in this work), the probe–oxide–semiconductor system forms a nanosized metal–oxide–semiconductor (MOS) structure. During sample scanning, changes in the capacitance of this structure in response to the applied accelerating voltage are measured. This enables the local electrical properties of the semiconductor layers to be determined, including the type of conductivity, relative changes in the carrier concentration, material composition, and the existence of localized electrical charge, similar to in classical C-V measurements [15]. However, due to the very small area of the MOS structure, and thus, its low structure capacitance values, the SCM output signal is proportional to the rate of capacitance change caused by the voltage change, denoted as dC/dV_AMP_. This amplitude is detected by the UHF capacitance sensor and corresponds to the slope of the dC/dV_AMP_ curve. In this way, the amplitude of the SCM signal is reversely proportional to the concentration of carriers in the sample, and its sign depends on the type of carriers: a plus sign corresponds to carriers with negative charges (electrons), and minus sign corresponds to carriers with positive charges (holes). An additional DC voltage (V_DC_) applied to the sample may be required to achieve flat-band conditions to maximize the SCM signal [16,17].

So far, SCM has successfully been employed to characterize electrical properties at the nanoscale in various cases, including GaN-based HEMT [18], 4H-SiC MOSFET transistors [19], electric field and carrier distribution in multi-quantum well GaAs/In0.2Ga0.8As p-i-n diodes [20], local currents and charge carrier distribution at the edges of GaAs p-n junction mesas after selective regrowth etching [21], correlating Ga donor concentration in ZnO determined by SIMS with the dC/dV signal obtained from SCM measurements [22], and observing doping transitions from p-type, through intrinsic, to n-type in PtSSe flakes [23]. In the field of photovoltaics, so far, SCM has been used to determine electrical junction positions in GaAs-based devices [24] and Cu(In,Ga)Se2 and Cu2ZnSnSe4 solar cells [25], to visualize carrier distribution profiles in perovskite solar cells [26], to image the potential-induced degradation influence on charge carrier distribution in multi-crystalline Si cells [27], to cross-check deep-level transient and admittance spectroscopies in perovskite solar cells [28], and to obtain dC/dV profiles in HgCdTe diodes corresponding to hole and electron profiles [29].

There are also reports in the literature on performing SCM measurements with simultaneous illumination of the sample (using white or monochromatic light), allowing for the determination of non-equilibrium carrier distribution in optoelectronic structures [30,31]. Our developed scanning capacitance microscopy measurement methodology with a unique option for controlled-wavelength illumination allows for both: obtaining SCM images/profiles of a sample excited using defined photon energy, and measuring the SCM signal vs. photon energy spectra at a specific location on the sample. This allows for nanoscale investigation of any optically excited energetic transition, which can provide information about compound semiconductor alloy composition, the existence of local stresses in structures, and deep energy-level defects [12].

The developed method is an adaptation of the macroscale steady-state photocapacitance spectroscopy method first described by Furukawa [32], which is used to study the electrical properties of various semiconductors [33,34]. The capacitance of the depletion region of the M-O-S structure is controlled by the space charge; therefore, any additional charge, generated by the optical population/depopulation of deep traps or interface states, as well as band-to-band generation, changes the thickness of the space-charge layer, and thus, the capacitance. Capacitance variations can be detected by the SCM sensor module, as in the classic measurement.

The optical system, integrated with an AFM microscope, consisted of a 250 W halogen illuminator, an automated Jobin Yvon 250 HR monochromator, an 800 nm bandpass filter, lenses, and a silica-core fiber with a 1000 μm diameter. This allowed for illumination of the sample over a wavelength range from 800 nm to 1600 nm with a resolution of 0.1 nm. The output beam optical power density was about 50 µW/cm^2^ at a 1000 nm wavelength. The measurement method is presented in Figure 1b. During scanning, the sample was illuminated by a beam of light coming from an optical fiber, positioned in such a way to ensure that the light beam was not shadowed by the microcantilever with an AFM tip. The SCM signal distribution profiles were measured on the side of the mechanically cleaved sample, perpendicular to its surface. One SCM scan line was measured at a specific optical excitation wavelength. Then, the wavelength was decreased by 0.5 nm, followed by a 10-s delay before the next scan line was recorded. This scheme was repeated over the range from 1600 nm to 800 nm (Figure 1c). The sample was placed in a metal holder coated with a layer of GaIn eutectic mixture to enable bottom electrical contact. The sample was scanned at a speed of 1 µm/s with force of about 200 nN applied to the tip, and the alternating voltage amplitude (V_AMP_) was set to 2000 mV. A Commercial NanoWorld PIT model AFM silicon probe was used, coated with an electrically conductive PtIr layer with a nominal tip radius <25 nm.

## 3. Results and Discussion

**Figure 2:**
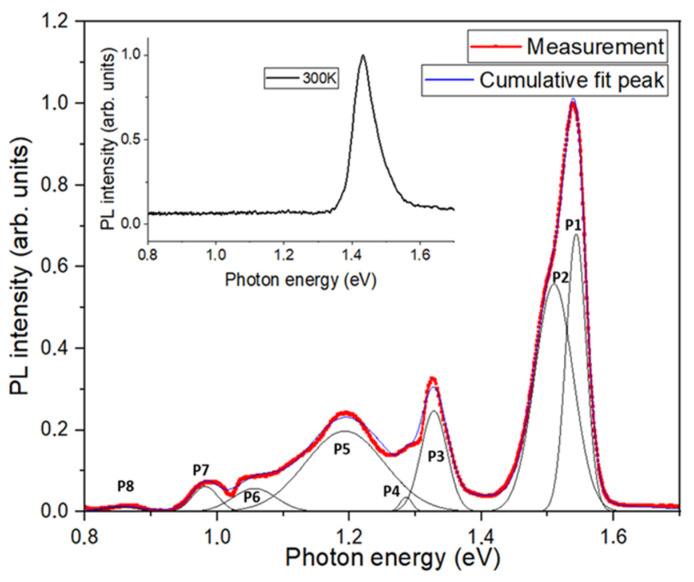
Low-temperature PL spectrum of GaAsN p-i-n solar cell structure measured at 90 K. Inset shows PL signal recorded at room temperature. Gaussian peak fitting results are shown as individual peaks (black line) and cumulative peak values (blue line).

A low-temperature photoluminescence spectrum of the GaAsN p-i-n solar cell is presented in Figure 2. The most pronounced feature is the PL peak related to the GaAs contact layer, AlGaAs window layer, and GaAs emitter, which was fitted with two Gaussian functions centered at 1.51 eV and 1.54 eV. The explanation for the origin of the 1.54 eV peak energy value is not straightforward because it exceeds the bandgap energy of GaAs. We speculate that this effect is somehow related to the very high doping of the top three layers of the solar cell [35] and/or effects related to the GaAs/AlGaAs and AlGaAs/GaAs interfaces, which we cannot directly explain here.

Additionally, for energies below 1.4 eV, a wide and complex PL emission component appears, which is most likely related to the non-homogenous GaAsN layer. In this part of the spectrum, the most significant two peaks, P3 and P5, are visible at energies of 1.32 eV and 1.20 eV, respectively.

Based on the two main peak positions, and using the temperature and composition dependences of the bandgap in GaAs_x_N_1−x_ alloys presented in [36,37,38], the corresponding percentage contents of N atoms were estimated to be 0.71% and 1.91%, respectively.

The existence of two clearly distinguishable peaks could be related to the nonhomogeneous composition of the GaAsN epilayer, as revealed through X-ray diffraction measurements (including both diffraction curves and reciprocal space maps) presented in our previous work [39]. Good agreement between the simulated layer composition and thickness and the recorded diffraction curve was obtained when assuming the existence of two GaAsN sublayers: in the first 200 nm layer, the nitrogen content changed concavely from 1.2% to 1.67%, while the second sublayer consisted of a uniform layer with 1.67% N and a thickness of about 600 nm. The nitrogen content inhomogeneity partially reduced the crystal lattice strain caused by the incorporation of nitrogen atoms at the As sites in GaAs. The difference in the estimated N content between X-ray diffraction and the PL measurement arises from their different methodologies. XRD detects signals from the total volume of the sample, while PL emissions are related to the recombination process from low-energy localized states.

It is also worth mentioning that the significant broadening of the P5 peak and the presence of the P4 peak at 1.29 eV indicate that the two-sublayer model of the GaAsN layer is too simplified and does not completely describe its compositional structure.

In addition to the aforementioned peaks, additional, less significant peaks (P6-P8) appear at lower energies. These are fitted with Gaussian peaks centered at 1.06 eV, 0.98 eV, and 0.85 eV. Multiple optical transitions present in the GaAsN photoluminescence spectra are commonly attributed to defects in the layers, caused by localized states induced by strong compositional inhomogeneity in the solid solution [40] and/or the incorporation of non-uniform nitrogen into the GaAs crystal lattice [41]. This non-uniformity results from significant differences in the sizes and electronegativity of the atoms of nitrogen and arsenic [41,42]. The appearance of additional peaks related to the GaAsN layer grown epitaxially on the GaAs substrate was also explained by the creation of a nitrogen-rich nanometer layer, a few nanometers thick, at the GaAsN/GaAs interface [42]. As can be seen in the inset of Figure 2, the photoluminescence from the GaAsN layer was not observed at room temperature. The weak RT luminescence of the as-grown GaAsN and the recovery of PL intensity after thermal annealing of these epilayers were reported in the literature [43] and were also observed in our GaAsN samples (not reported here).

**Figure 3:**
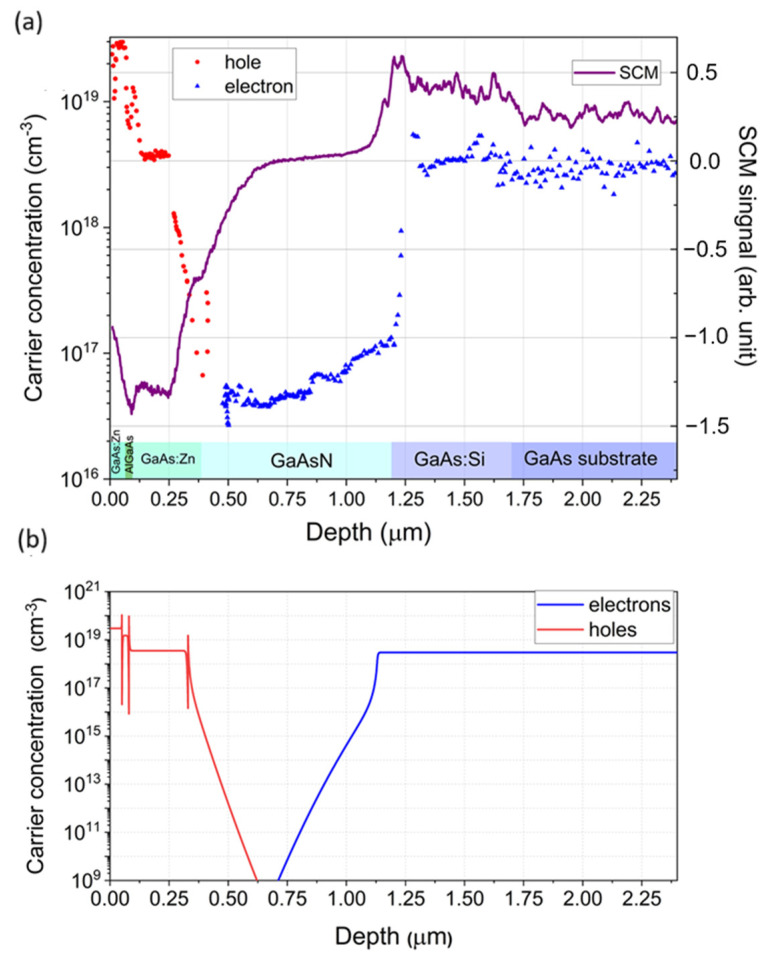
Distribution of free carrier concentration measured using E-CV (left axis) and SCM signal (right axis) across GaAsN p-i-n solar cell structure (**a**); calculated theoretical concentrations of electrons and holes (**b**).

A comparison of the electrical characterization results of the samples using the EC-V and scanning capacitance microscopy methods is presented in Figure 3a. Both methods allow for defining the position and thickness of the individual layers in the structure and provide some information about electrically active carriers. The EC-V method also provides quantitative results regarding the concentration of individual types of current carriers, while in the case of the SCM method, they are visible only as relative changes in the signal value. The carrier concentration profiles differ in shape for these two methods due to their different measurement configuration schemes. In the case of EC-V measurements, the carrier concentration is sampled from the top surface of the sample by inducing a depletion zone through electrolyte–semiconductor contact. Thus, this method senses the properties of the layer located directly under the Schottky-like contact.

In SCM measurements, the AFM microscope tip scans the cross-section side of the structure, determining the carrier concentration at specific points. This reflects both the doping of the layers and the influence of internal electric fields (in this case, those created by the p-i-n junction). Therefore, the SCM profile is highly consistent with the free carrier concentration profile obtained as a result of the numerical simulation of our p-i-n structure carried out using SimWindows version 1.5 software [44] and shown in Figure 3a. SimWindows is a 1D device simulator developed by Dave Winston at the University of Colorado. It numerically solves the coupled differential transport, continuity, and Schrödinger–Poisson equations. The simulation and SCM measurement profiles also show agreement in determining the electrical position of the p-i-n junction, indicated by the SCM signal passing through the zero value at about 0.7 µm.

**Figure 4:**
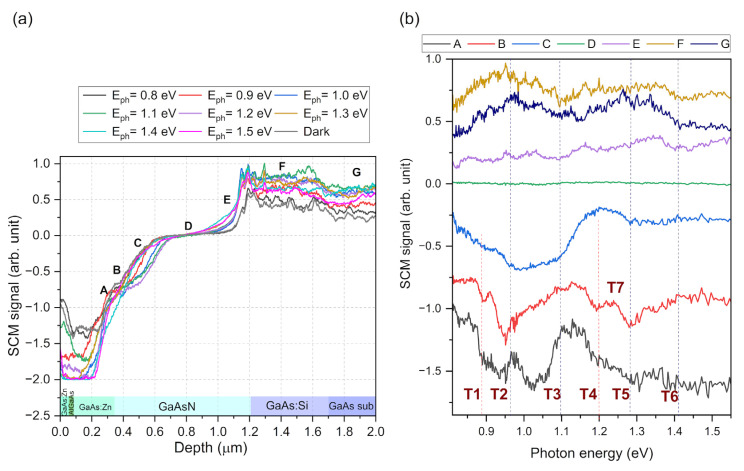
SCM signal across GaAsN p-i-n solar cell structure measured for different illumination photon energies (**a**); photo-SCM signal spectra measured at different locations of the cross-section of the GaAsN p-i-n solar cell structure (**b**).

In order to perform an in-depth investigation of the effects occurring in the p-i-n solar cell structure with a GaAsN base, SCM measurements were carried out with simultaneous illumination of the sample. The SCM signal distribution profiles for the different photon energies of light used to excite the sample are shown in Figure 4a. The observed signal represents the current carrier emission behavior related to energetic states existing in the semiconductor material. In general, a visible increase was observed in the signal for both holes on the p-type side and electrons on the n-type side of the junction. This was caused by the separation of these carriers by the junction’s built-in electric field. In the middle of the junction, the observed changes were minimal because the electric field is the highest in this area, effectively sweeping away the photo-generated carriers. To investigate the properties of individual parts of the structure, spectra showing the dependence of the SCM signal on the energy of the exciting photons were obtained. Figure 4b presents the spectra measured at specific depth positions along the profile, marked as A to G in Figure 4a. Energy transitions appear a recognizable thresholds in the spectrum graph, visible as peaks, valleys, or kinks.

Figure 4b shows seven significant energy transitions at the following photon energies: T1 = ~0.89 eV, T2 = ~0.96 eV, T3 = ~1.10 eV, T4 = ~1.20 eV, T5 = ~1.28 eV, and T6 = ~1.42 eV. The signal change attributed to the gallium arsenide bandgap at 1.42 eV is most prominent in the spectra measured at the GaAs buffer and substrate regions (F and G). The spectrum of the signal measured in the junction center, labeled D has, remained constant for all photon energies, further indicating the effective sweeping of carriers from this area. In spectrum E, measured in the junction area on the GaAs substrate side, many energy transitions are evident, but the relative magnitude of changes is small. The T1 and T4 transitions are most visible on the side of the junction area closer to top GaAs region (A, B, C). The most dominant transitions are T2 and T4, which appeared in all areas of the tested structure. It is also worth noting the peak occurring in spectrum A, at a photon energy of about 1 eV; since it only occurs close to the sample surface, it likely arises from energy levels associated with surface states. A characteristic feature of position B is the presence of a distinct T7 (~1.25 eV) transition, which appeared only in this case.

According to the results in Figure 4, it can be concluded that the presence of the p-i-n junction electric field influences the measured SCM spectra. To investigate the properties of only GaAsN, SCM measurements of a single GaAsN epilayer grown on a GaAs substrate were performed. XRD structural characterization (not presented here) also confirmed that the good agreement between the measured diffraction curve and the curve simulated using dynamic diffraction theory could be achieved by modeling the GaAsN layer with two sublayers with different N contents: one 210 thick with a gradient N content ranging from 0.9 to 1.48%, and the second 610 nm thick with 1.48% constant N content.

**Figure 5:**
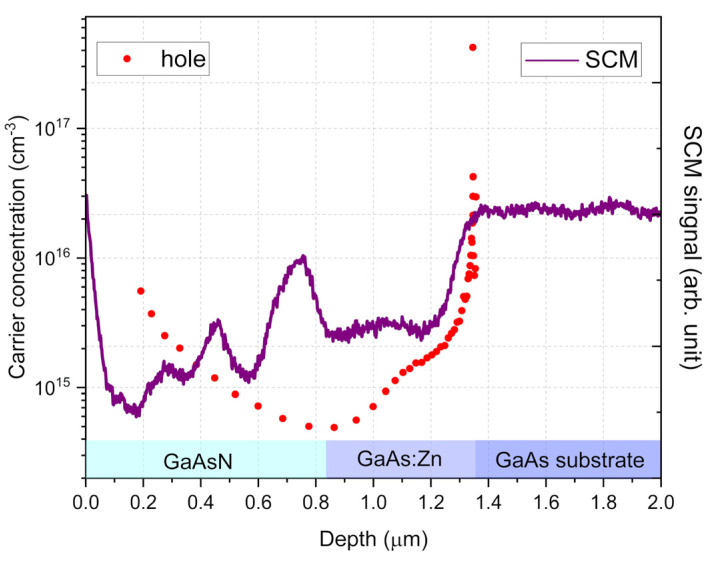
Distribution of free carrier concentration measured by EC-V (left axis) and SCM signal (right axis) across GaAsN/p-GaAs:Zn/p-GaAs substrate structure.

**Figure 6:**
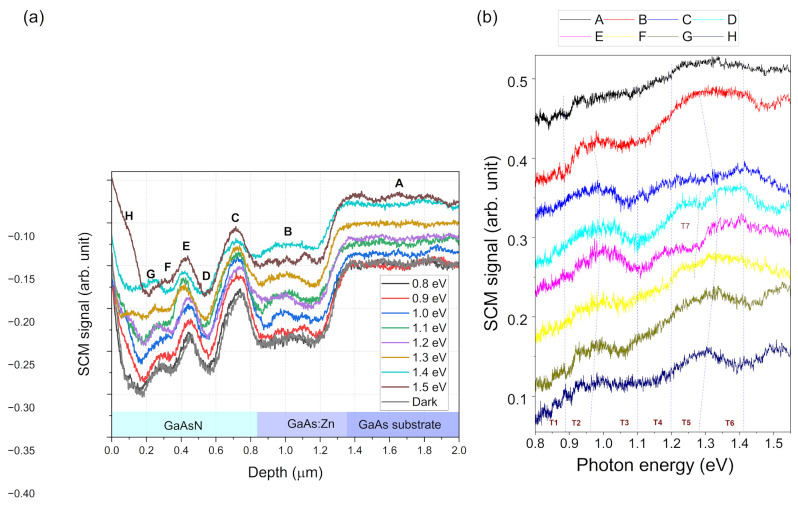
SCM signal across GaAsN/p-GaAs:Zn/p-GaAs substrate structure measured at different illumination photon energies (**a**); photo-SCM signal spectra measured at different locations of the cross-section of the GaAsN/GaAs:Zn/GaAs substrate structure (**b**).

The results of electrical characterization using the EC-V and SCM methods on the test sample consisting of 820 nm thick GaAsN and 450 nm thick zinc-doped p-GaAs:Zn buffer grown on p-type GaAs substrate are presented in Figure 5 (note: in Figure 5 and Figure 6, the SCM signal has been shifted and scaled to highlight the characteristic features).

The SCM method allows for distinguishing and determining the thickness of individual layers with much greater resolution. The resolution of the EC-V technique is determined by the depletion layer width of the Schottky barrier created by the electrolyte–semiconductor contact. This width is inversely proportional to the carrier concentration. In the present case of the undoped GaAsN layer (carrier concentration ~10^15^ cm^−3^), the width is in the order of hundreds of nm. This means that the interfaces between layers with different carrier concentrations are not clearly distinguishable. It also explains why the first concentration measurement point in the E-CV profile is located at a depth of approximately 200 nm: it corresponds to the initial penetration depth of the depletion zone during the first measurement. Simultaneously, the resolution of the SCM measurement primarily depends on the size of the contact of the microscope probe, defined in this case by the AFM tip radius, and is less affected by the concentration of carriers in the semiconductor. In this way, it is possible to distinguish the components of the measured structure in the SCM profile as significant signal changes at the boundaries between layers.

The carrier concentration distribution obtained using the EC-V method is consistent with the expectations and previous measurements for solar cells, i.e., a very low concentration was observed for the undoped GaAsN layer and an increase in these values was observed for the doped layers of p-type GaAs:Zn buffer layer and GaAs substrate. In the case of the SCM measurements, the profiles for both GaAs layers were also as expected, i.e., the absolute value depended on the doping concentrations. However, unusually, the depth profile of the SCM signal exhibits significant magnitude fluctuations across the GaAsN layer. Since the E-CV measurements prove that this variation cannot be caused by changes in carrier concentration, it can be assumed that they resulted from the material inhomogeneity of the GaAsN layer. This was expected based on the PL measurements presented in Figure 2 (as well as the literature reports cited earlier).

Similarly to the solar cell structure, SCM measurements with simultaneous illumination were performed on the sample with a single GaAsN layer. The signal profiles (Figure 6a) and spectra (Figure 6b) at specific depth locations (depicted as letters A to H in Figure 6a) were determined. In general, it is clear that energy transitions occurred at similar energies as those observed for the GaAsN layer as a component of the solar cell.

The spectrum shown in Figure 6b is very extensive, which indicates the occurrence of many energy transitions related to the defects and inhomogeneities of the GaAsN layer structure, similarly to the photoluminescence spectrum shown in Figure 2. However, directly comparing the individual features visible in these two spectra would be unjustified because they result from two different phenomena present in the illuminated semiconductor. In the PL method, results related to the radiative recombination of carriers from specific energy levels were observed. In the case of the SCM method, changes in electric charge were observed as a result of optical generation. The energy levels at which these transitions occur may be the same, but do not necessarily have to be, for both described phenomena.

In the analysis of photocapacitance spectra, the shape of characteristic changes in the spectrum curve resulting from the energy transition at a specific photon energy indicates the distribution of the energy state levels associated with this transition. For example, a narrow peak in the spectrum or a sharp increase in the signal at a specific photon energy indicates a transition associated with discrete energy states. When these features are broadened, it indicates a wider distribution of energy levels involved in this transition. In the photo-SCM spectroscopy case presented here, detailed analysis of the spectral curve shape is very difficult because, despite the very small sampling area of the microscope tip, the SCM signal is distorted by the influence of carriers diffusing optically in other parts of the structure. This is because, as shown in the SCM measurement scheme (Figure 1b), the entire area of the structure cross-section is illuminated during the measurement. In this case, carriers excited in other places of the sample may diffuse, affecting the signal measured at a specific location. For the diffusion length in GaAsN defined at about 400 nm [45], this could occur throughout a significant part of the GaAsN layer volume. The diffusion effect also means that the area from which the spectrum is collected is not determined solely by the size of the AFM microscope tip, reducing the resolution of the method. Despite this, it was possible to correlate some specific transition energy levels with certain areas or depths of the studied structure, based on the clarity and magnitude of specific features.

Based on the observed dependence of specific energy transitions on their depth in the GaAsN layer, two types of transitions can be distinguished: those that occur at a specific location/depth of the layer and those that occur throughout its volume. One of the first type of transitions is T7, at 1.25 eV, whose marker is only visible at depths from 320 nm to 520 nm. The T1 (0.89 eV) transition also has a defined location, which occurs near the interface with the Zn-doped GaAs layer in the case of both the p-i-n cell structure and the single GaAsN layer. Interestingly, in the case of the single GaAsN layer, the signature of this transition is much more visible on the side of the GaAs layer.

The T2, T3, and T5 transitions belong to the group observed throughout the entire cross-section of the GaAsN layer. T3 appears at a constant energy of 1.1 eV in all measured spectra and, therefore, can be related to the formation of (AsN)_As_ complexes. According to [46], these complexes are energetically fixed at about E_V_ + 1.1 eV above the valence band.

A characteristic feature of the T2 and T5 transitions is the shift in their peak energies depending on the depth at which they occur, visible as a bending of the line connecting their centers in Figure 6b. The energy of the T5 transition, which is attributed to a band-to-band transition, varies from 1.27 to 1.33 eV, which may be caused by changes in the N concentration in GaAsN. The changes in the T2 peak energy position as a function of depth within the layer reflect those observed for T5, and appear at about 0.3 eV below the T5 energy. This suggests that this transition may be related to the N-related deep level ((N-N)_As_ or (N-As)_As_), which has been identified as forming an energy level about 0.3 eV below the bottom of the GaAsN conduction band [47].

The T4 transition is the most difficult to define, as it is unclear if it is characteristic of a specific location in the layer or it if occurs throughout its cross-section. The strongest sign of this transition occurs near the center of the GaAsN layer, but weak signals are also reflected at other positions, perhaps as a result of the carrier diffusion effect.

Finally, the T6 transition, which is attributed to the gallium arsenide bandgap and induced through optical generation in the GaAs substrate and buffer layer, is visible at all depths of the GaAsN layer. This is due to the much larger volume of these layers in relation to the GaAsN layer, and the resulting large number of photogenerated carriers diffusing into the neighboring layer.

## 4. Conclusions

The developed investigation methodology, combining scanning capacitive microscopy with tunable light excitation, allowed for imaging of characteristic properties in p-i-n solar cell device structures, such as the actual dimensions of individual layers, the distribution of carriers in the semiconductor junction, and the location of the junction center. Measurements focused on the epitaxial GaAsN layer supplement and extend findings obtained from photoluminescence studies of the layers and a previously performed structural characterization of the layers using the XRD technique. Both PL and photo-SCM revealed a complex structure of optically generated transitions, related to the structural inhomogeneity of the GaAsN layers. However, the latter method allowed for obtaining information on the spatial location of individual energy transitions within the depth of the profile cross-section. Some transitions were associated with a specific location: in the middle of the structure depth (T7: 1.25 eV and probably T4: 1.2 eV) or near the GaAsN/p-doped GaAs interface (T1: 0.89 eV). Other transitions were associated with N-related defects extending uniformly throughout the entire depth of the layer (T3: 1.1 eV and T2: 0.97–1.03 eV). An analysis of the change in depth of the T5 peak position (1.27–1.33 eV) in the photo-SCM spectra, associated with the energetic band–band transitions, confirms the conclusion from XRD studies regarding the existence of nitrogen content changes in the GaAsN layer. However, this indicates that this gradient change in nitrogen content occurs continuously throughout the entire depth of the layer and is not limited to the 200 nm thick sublayer.

The presented method of measuring the opto-electrical properties of semiconductor structures and materials with a resolution of tens of nanometers has unique possibilities compared to other methods of material characterization. Compared to methods such as PL, deep-level transient spectroscopy, and X-ray diffraction, which average values from a certain sample volume, this method allows for sampling the local properties of the structures. Methods such as transmission electron microscopy, energy-dispersive spectroscopy, and secondary-ion mass spectrometry have high resolution, but they provide information on the structural/chemical properties of the material, not the electrical ones, which are most important for device structure development. A method that enables the examination of the electro-optical properties of layers with a spatial resolution similar to the photo-SCM method presented here is cathodoluminescence; however, it requires maintaining a vacuum during the measurements. An additional advantage of this photo-SCM method is a relatively inexpensive and simple way of conducting the measurement: simple sample preparation, measurements performed in air and at room temperature.

## Figures and Tables

**Table 1:** Growth parameters of investigated samples.

Growth Parameter	GaAsN p-i-n Solar Cell	GaAsN
V/III ratio for GaAs	67.76	67.76
V/III ratio for GaAsN	59.87	59.87
V/III ratio for AlGaAs	12.12	-
SiH4 flow (ml/min)	100	-
DEZn flow (ml/min)	2 (GaAs emitter)	-
	100 (AlGaAs)	-
	100 (GaAs contact layer)	-
	-	100 (GaAs buffer)

## Data Availability

The raw data supporting the conclusions of this article will be made available by the authors upon request.

## Abbreviations

The following abbreviations are used in this manuscript:

AFM	Atomic Force Microscopy
AP-MOVPE	atmospheric pressure metal-organic vapour phase epitaxy
EC-V	Electrochemical Capacitance-Voltage
PL	Photoluminescence
SCM	Scanning Capacitance Microscopy
UHF	Ultra High Frequency
